# Supplementary material for: Estimating small area population from health intervention campaign surveys and partially observed settlement data
Source: Nat Commun. 2025 May 28;16:4951. doi: 10.1038/s41467-025-59862-4 (PMC12119932; doi:10.1038/s41467-025-59862-4)
Supplement: Supplementary file 1 — Supplementary Information [file 41467_2025_59862_MOESM1_ESM.pdf]

Estimating small area population from health intervention campaign surveys and settlement data

Chibuzor Christopher Nnanatu[1,2]*, Amy Bonnie[1], Josiah Joseph[3], Ortis Yankey[1], Duygu Cihan[1], Assane Gadiaga[1], Hal Voepel[1], Thomas Abbott[1], Heather Chamberlain[1], Mercedita Tia[4], Marielle Sander[4], Justin Davis[5], Attila Lazar[1] and Andrew J. Tatem[1]

[1]WorldPop, School of Geography and Environmental Science, University of Southampton, Southampton, UK

[2]Nnamdi Azikiwe University, Nigeria

[3]National Statistical Office, Papua New Guinea

[4]United Nations Population Fund, Papua New Guinea

[5]Planet Labs, San Francisco, USA

*Corresponding Author's email: cc.nnanatu@soton.ac.uk

SUPPLEMENTAL MATERIALS

Simulation Study Results

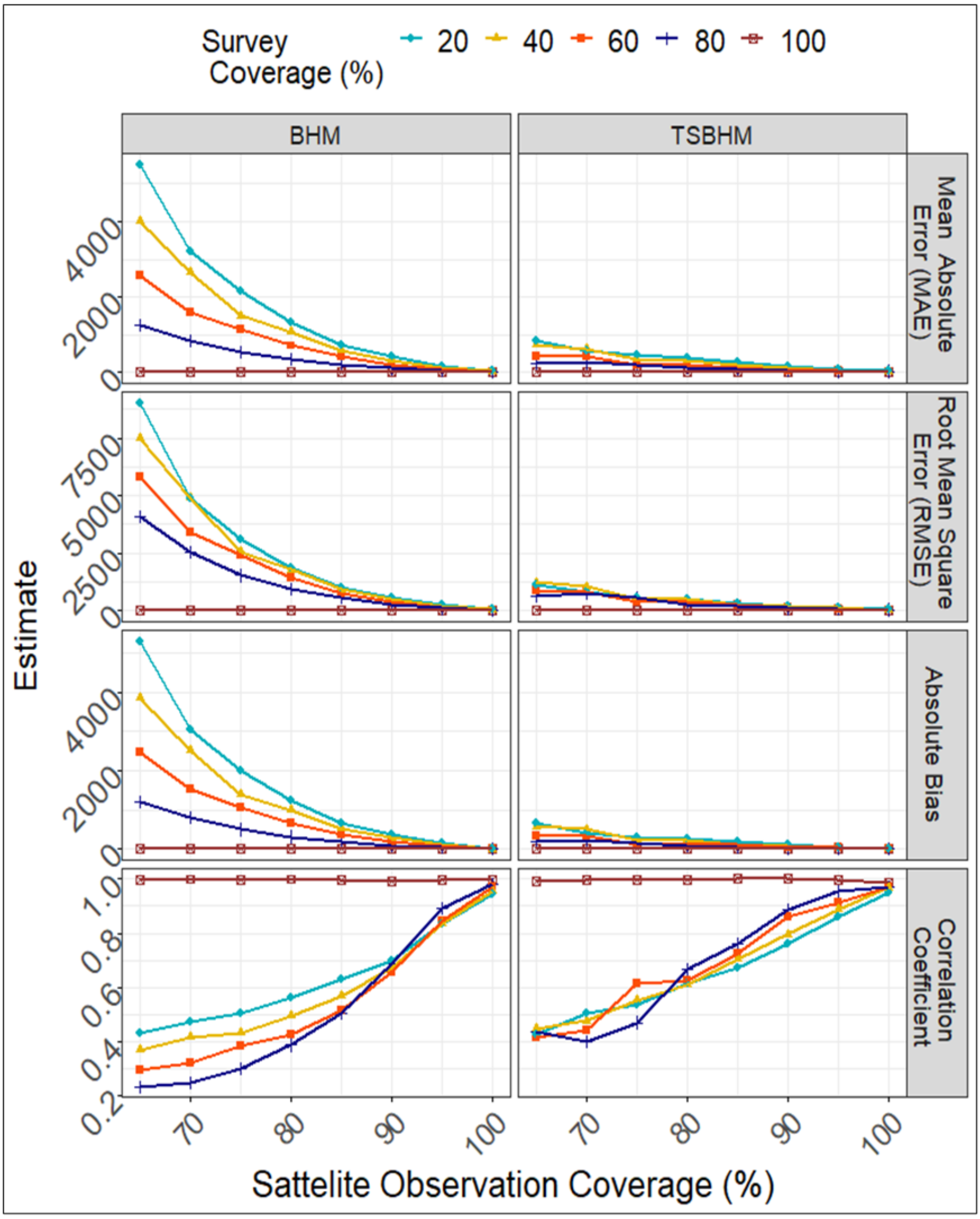

Figure S1. Simulation study model fit metrics. The figure shows that the TSBHM approach provided the best fits across all scenarios.

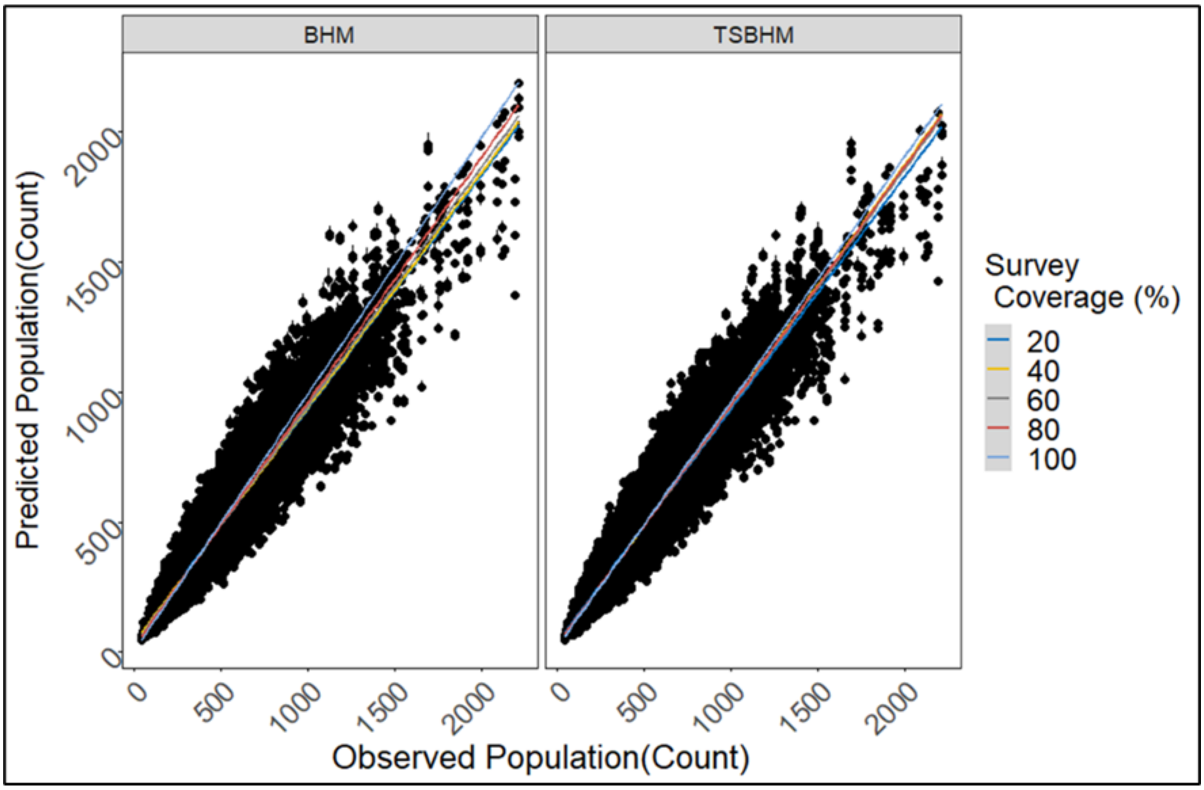

Figure S2: Scatter plots of simulated (observed) versus predicted population counts based on the TSBHM and BHM approaches across various proportions of survey coverage when the settlement data are completely observed. Both models performed well when all the settlement data were fully observed. However, the BHM approach became much more uncertain in estimation as the proportion of unsurveyed locations increased.

Papua New Guinea (PNG) application Results

Table S1. Posterior Model Parameter Estimates of the best fit models for both BHM and TSBHM

Variable/Effects Description	Mean		SD		95%CI (Lower)		95%CI (Upper)	
	BHM	TSBHM	BHM	TSBHM	BHM	TSBHM	BHM	TSBHM
Intercept	-5.8859	-5.9569	0.2198	0.2032	-6.3237	-6.3572	-5.4467	-5.5561
cov1	-0.0151	-0.0478	0.0343	0.0247	-0.0825	-0.0964	0.0522	7.00E-04
cov2	-0.027	-0.0007	0.0186	0.0126	-0.0635	-0.0254	0.0095	0.0239
cov3	-0.01	0.0267	0.0184	0.0123	-0.0461	0.0025	0.026	0.0509
cov5	0.0469	0.0655	0.0403	0.0308	-0.0322	0.0051	0.1259	0.1259
cov6	0.0838	0.0842	0.0194	0.0138	0.0457	0.0571	0.1217	0.1114
cov7	0.2900	0.3614	0.0455	0.0349	0.2010	0.293	0.3794	0.4299
cov8	-0.1907	-0.1906	0.053	0.0453	-0.2949	-0.2794	-0.0868	-0.1017
cov11	0.0226	0.0562	0.0241	0.0163	-0.0247	0.0241	0.0699	0.0883
cov12	-0.0469	-0.0509	0.0248	0.0182	-0.0956	-0.0866	0.0018	-0.0153

cov14	0.0301	-0.0063	0.0187	0.0125	-0.0066	-0.0309	0.0668	0.0182
cov15	0.0082	0.0025	0.032	0.0261	-0.0542	-0.0487	0.0714	0.0539
cov16	0.2669	0.2927	0.0702	0.0699	0.1283	0.1559	0.4045	0.4303
cov17	-0.0112	0.0064	0.0138	0.0092	-0.0383	-0.0116	0.0158	0.0243
cov19	-0.0999	-0.1357	0.012	0.0078	-0.1234	-0.151	-0.0764	-0.1205
cov20	0.2489	0.2603	0.0177	0.0118	0.2143	0.2371	0.2836	0.2835
$\frac{1}{\sigma_\epsilon}$	438.2548	509.0265	186.32	192.7036	204.6822	195.7346	914.056	934.7377
τ_ξ	0.5567	1.288	0.0062	0.0143	0.5449	1.2597	0.5691	1.3157
τ_{setTyp}	20.3876	24.7686	8.296	8.0951	7.2819	11.0299	39.2599	42.3759
σ_c^2	0.8345	0.8621	0.1235	0.0952	0.6345	0.6898	0.8187	0.8568

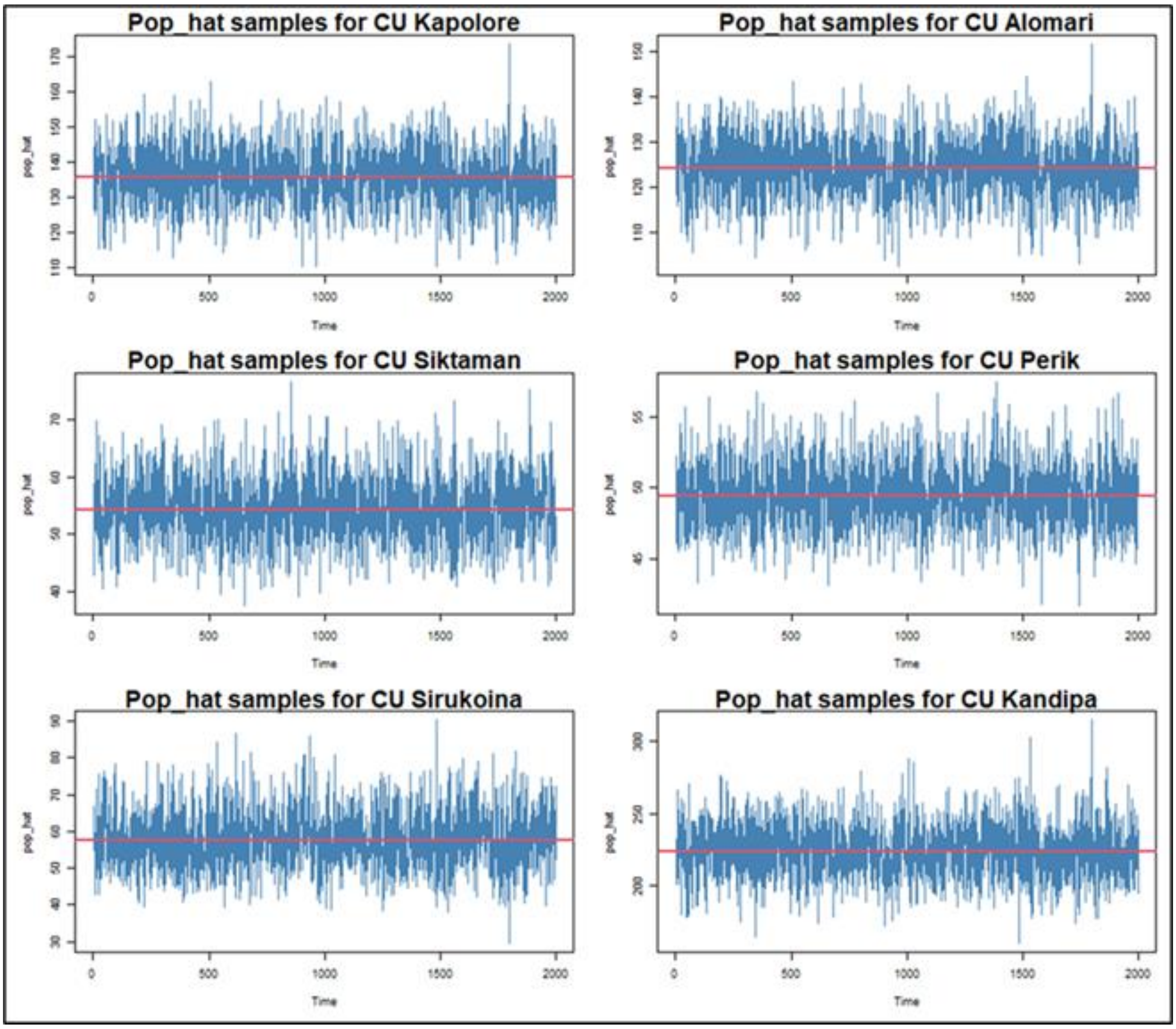

Figure S3: Trace plots of the posterior samples of six randomly selected census units after taking a burn-in period of 20%. The samples were mixing well, indicating that the posterior samples were taken from the target probability distribution.

Methods

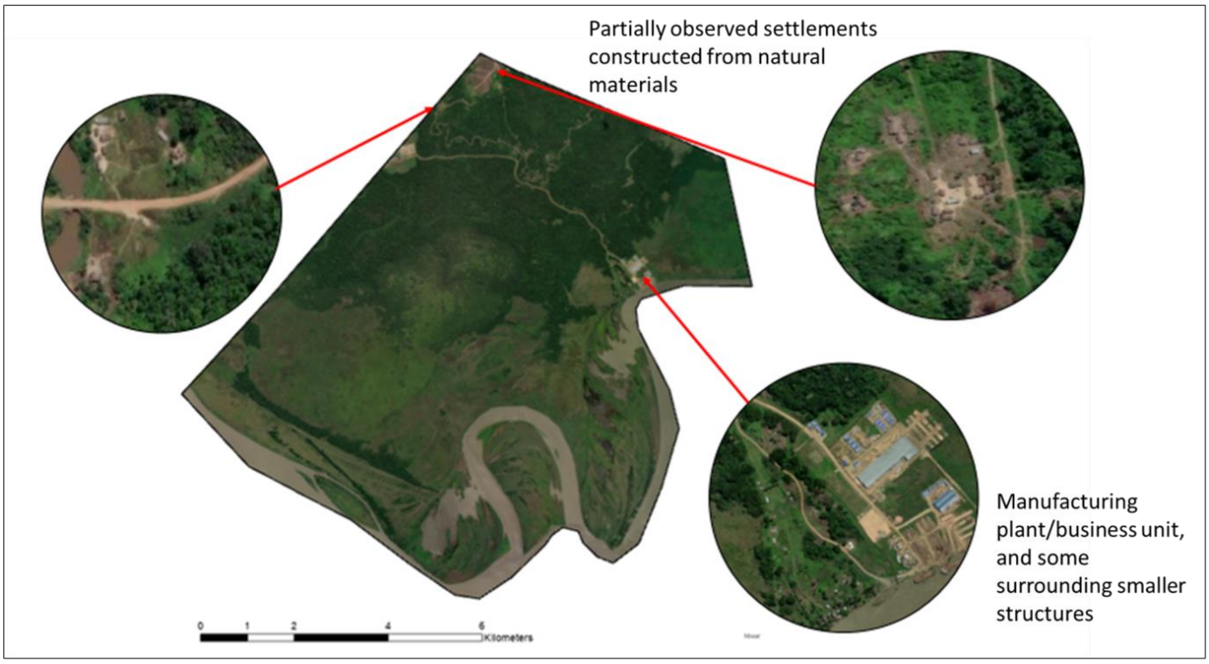

Figure S4. Comparisons of recent high resolution satellite imagery (sourced from ESRI) against the 2019-21 malaria survey data within administrative unit Kanduanum 2.

Figure S4 presents an example of patterns seen across rural areas of Papua New Guinea (and is also common to other tropical countries). Here, the satellite imagery shows some small areas of settlement, but an insufficient number of residential buildings are visible to house the 891 people recorded by the 2019-21 malaria survey as residing in the administrative unit, with the remainder of structures hidden under dense tree canopy

Table S2: The final model covariates selected via stepwise regression.

Variable	Description	Data type	Population density model	Building intensity model
cov1	Mean total daily precipitation	Continuous	✓	✓
cov2	Baseflow Index 1	Continuous	✓	-
cov3	Baseflow Recession	Continuous	✓	✓
cov4	Motorized friction surface	Continuous	-	✓
cov5	Distance to health providers	Continuous	✓	-
cov6	Distance to local roads	Continuous	✓	✓

cov7	Distance to main roads	Continuous	√	√
cov8	Distance to marketplace	Continuous	√	-
cov9	Distance to places of education	Continuous	-	√
cov10	Distance to places of worship	Continuous	-	√
cov11	Distance to aquatic vegetation areas	Continuous	√	√
cov12	Distance to artificial surface edges	Continuous	√	√
cov13	Distance to cultivated areas	Continuous	-	√
cov14	Distance to ESA-CCI-LC inland water	Continuous	√	-
cov15	Distance to OSM major waterways	Continuous	√	-
cov16	Distance to shrub area edges	Continuous	√	√
cov17	Distance to woody areas	Continuous	√	-
cov18	Resampled DMSP-OLS night-time lights	Continuous	-	√
cov19	Resampled VIIRS night-time lights	Continuous	√	-
cov20	Slope	Continuous	√	√

Note: These are the final geospatial covariates retained across the best fit geospatial models. For the two-step modelling approach,15 covariates were selected for the population density model, while 13 covariates were selected for the building intensity/building count model. See Table S3 below for more descriptions and sources of the model covariates.

Table S3. Description and Sources of the model covariates (mostly time-invariant covariates)

Covariate	Date	Unit	Source	Link
Slope	2000	Degrees	WorldPop	https://www.worldpop.org/geodata/summary?id=23186
Elevation	2000	Metres	WorldPop	https://www.worldpop.org/geodata/summary?id=23435
Resampled VIIRS night-time lights	2016	nanoWatts/cm2/sr	WorldPop	https://www.worldpop.org/geodata/summary?id=18704
Distance to IUCN strict nature reserve and wilderness area edges	2017	Kilometres	WorldPop	https://www.worldpop.org/geodata/summary?id=18215
Resampled DMSP-OLS night-time lights	2011	Unit of radiance ranging from 0-6300	WorldPop	https://www.worldpop.org/geodata/summary?id=18953

Distance to open-water coastline	2020	Kilometres	WorldPop	https://www.worldpop.org/geodata/summary?id=23933
Distance to ESA-CCI-LC inland water	2012	Kilometres	WorldPop	https://www.worldpop.org/geodata/summary?id=24182
Distance to cultivated areas	2015	Kilometres	WorldPop	https://www.worldpop.org/geodata/summary?id=22937
Distance to woody areas	2015	Kilometres	WorldPop	https://www.worldpop.org/geodata/summary?id=22937
Distance to shrub area edges	2015	Kilometres	WorldPop	https://www.worldpop.org/geodata/summary?id=22937
Distance to herbaceous areas	2015	Kilometres	WorldPop	https://www.worldpop.org/geodata/summary?id=22937
Distance to sparse vegetation areas	2015	Kilometres	WorldPop	https://www.worldpop.org/geodata/summary?id=22937
Distance to aquatic vegetation areas	2015	Kilometres	WorldPop	https://www.worldpop.org/geodata/summary?id=22937
Distance to artificial surface edges	2015	Kilometres	WorldPop	https://www.worldpop.org/geodata/summary?id=22937
Distance to bare areas	2015	Kilometres	WorldPop	https://www.worldpop.org/geodata/summary?id=22937
Distance to OSM major road intersections	2016	Kilometres	WorldPop	https://www.worldpop.org/geodata/summary?id=17717
Distance to OSM major waterways	2016	Kilometres	WorldPop	https://www.worldpop.org/geodata/summary?id=17966
Distance to OSM major roads	2016	Kilometres	WorldPop	https://www.worldpop.org/geodata/summary?id=17468
Distance to main roads	2016-2021	Decimal degrees	OSM	https://download.geofabrik.de/australia-oceania/papua-new-guinea.html
Distance to local roads	2016-2021	Decimal degrees	OSM	https://download.geofabrik.de/australia-oceania/papua-new-guinea.html
Distance to places of worship	2016-2021	Decimal degrees	OSM	https://download.geofabrik.de/australia-oceania/papua-new-guinea.html
Distance to places of education	2016-2021	Decimal degrees	OSM	https://download.geofabrik.de/australia-oceania/papua-new-guinea.html
Distance to health providers	2016-2021	Decimal degrees	OSM	https://download.geofabrik.de/australia-

				oceania/papua-new-guinea.html
Distance to marketplace	2016-2021	Decimal degrees	OSM	https://download.geofabrik.de/australia-oceania/papua-new-guinea.html
Motorized friction surface	2019	Minutes required to travel 1 metre	MAP	https://malariaatlas.org/explorer/#/
Walking friction surface	2019	Minutes required to travel 1 metre	MAP	https://malariaatlas.org/explorer/#/
Mean 2m dewpoint temperature	2011-2021	Celsius	Copernicus	https://cds.climate.copernicus.eu/cdsapp#!/dataset/reanalysis-era5-land-monthly-means?tab=form
Mean 2m temperature	2011-2021	Celsius	Copernicus	https://cds.climate.copernicus.eu/cdsapp#!/dataset/reanalysis-era5-land-monthly-means?tab=form
Mean total daily precipitation	2011-2021	Metres	Copernicus	https://cds.climate.copernicus.eu/cdsapp#!/dataset/reanalysis-era5-land-monthly-means?tab=form

Table S4: Description of key mathematical symbols and notations

Symbol/Notation	Data/Parameter/function	Description
B	Data	Observed building intensity/Building count
$\bar{b}$	Parameter	Average building intensity/Average building count
$\hat{B}$	Model prediction	Predicted building intensity/Predicted building count
D	Data	Observed population density
$\bar{d}$	Parameter	Average population density
$\hat{D}$	Model prediction	Predicted population density
C	Data	Population count
λ	Parameter	Average population
$\hat{C}$	Model prediction	Predicted population
$x_1, \dots, x_M$	Data	Geospatial covariates (e.g., distance to schools)
$z_1, \dots, z_L$	Data	Factor levels of categorical variables (e.g., Province)
$\boldsymbol{\beta} = (\beta_0, \beta_1, \dots \beta_M)$	Parameter	Unknown regression coefficients of the covariates
$\eta^{(.)}$	Parameter	Linear predictor
$g(.)$	Function	Link function
$\sigma^2_{(.)}$	Parameter	Variance parameter
$\tau = 1/\sigma^2_{(.)}$	Parameter	Precision parameter
$\boldsymbol{A}_{(.)}$	Parameter	Projection matrix that maps observations to mesh nodes

ξ	Parameter	Spatially varying random effects
ζ	Parameter	Nugget effect or spatially independent random effects
$\vartheta_{(.)}$	Parameter	Random intercepts for settlement types, Province, etc
$\alpha_1^{(.)}$	Parameter	Rate parameter of a Gamma density
$\alpha_2^{(.)}$	Parameter	Shape parameter of a Gamma density
ν	Parameter	Smooth parameter of co variance function
s	Data	Spatial location of a point
κ	Parameter	Scale parameter of a covariance function
$Cov(.,.)$	Function	Covariance between two points in space
$SPDE$	Function	Stochastic Partial Differential Equation framework
$GMRF$	Function	Gaussian Markov Random Field concept

Bayesian hierarchical modelling (BHM) framework for bottom-up population modelling

We assume that the observed population count C_i at spatial unit (census unit) i across the $n\ (= 32100)$ census units in PNG follows a Poisson distribution with mean $\lambda_i = \bar{d}_i \times B_i$, that is,

$$C_i|\lambda_i \sim Poisson(\bar{d}_i B_i) \qquad (S1)$$

where $\bar{d}_i$ is the mean population density and B_i is the number of buildings (human settlement structures) in census unit i. The nominal estimate of the population density D_i for each census unit in this context is given by the number of people per building, that is,

$$D_i = \frac{C_i}{B_i} \qquad (S2)$$

so that D_i is a non-negative (often right skewed) continuously distributed random variable, herein assumed to follow a Gamma probability distribution with shape and rate parameters $\alpha_1^{(D)}$ and $\alpha_2^{(D)}$. Thus,

$$D_i \sim Gamma\left(\alpha_1^{(D)}, \alpha_2^{(D)}\right) \qquad (S3)$$

with mean $E[D_i] = \bar{d}_i = \alpha_1^{(D)}/\alpha_2^{(D)}$ and variance, $var(D_i) = \sigma_D^2 = \alpha_1^{(D)}/(\alpha_2^{(D)})^2$.

We assume that the mean population density $\bar{d}_i$ depends on a set of geospatial covariates $\mathrm{x}_1, \dots, \mathrm{x}_K$ (e.g., night time light brightness, distance to market, etc) and other auxiliary variables $z_1, \dots, z_L$ (e.g., settlement type, spatial autocorrelation, etc) through the structured additive predictor $\eta_i^{(D)}$ given by

$$g(\bar{d}_i) = \eta_i^{(D)} = \beta_0 + \sum_{k=1}^{K} \beta_k \mathrm{x}_{ik} + \sum_{l=1}^{L} \vartheta_l z_{il} \qquad (S4)$$

where β_0 is the intercept parameter, which represents the baseline (average) population density when the effect of the other predictors is zero; $\boldsymbol{\beta} = \{\beta_1, \ldots, \beta_K\}$ is a vector of unknown fixed effects coefficients of the K geospatial covariates; ϑ_l $(l = 1, \ldots, L)$ is a vector of parameters corresponding to the random effect factors z_l $(l = 1, \ldots, L)$ with L levels such as settlement types, temporal or spatial random effects or random intercepts.

We used a log link function $g(.)$ so that $log(\bar{d}_i) = \eta_i^{(D)} = \beta_0 + \sum_{k=1}^{K} \beta_k \mathrm{x}_{ik} + \sum_{l=1}^{L} \vartheta_l z_{il}$ and the predicted population density is given by

$$\widehat{D}_i = \exp\left(\beta_0 + \sum_{k=1}^{K} \beta_k \mathrm{x}_{ik} + \sum_{l=1}^{L} \vartheta_l z_{il}\right) \qquad (S5)$$

that is, $\widehat{D}_i = \exp\left(\eta_i^{(D)}\right)$.

Spatial Autocorrelation

We extended equation (*S4*) to include a spatial random effects term. This became necessary since the population density across PNG is spatially heterogeneous, with some units having more similar spatial patterns than others. Therefore, there is need to account for the potential effects of spatial autocorrelation within the observations for a deeper understanding of the spatial distribution of population density. In addition, the integration of spatial autocorrelation within the modelling framework means that we can 'borrow strength' from census units with observations to predict estimates of population counts at contiguous census units with few or no observations. Thus,

$$g(\bar{d}_i) = \eta_i^{(D)} = \beta_0 + \sum_{k=1}^{K} \beta_k \mathrm{x}_{ik} + \sum_{l=1}^{L} \vartheta_l (z_{il}) + \xi_i \qquad (S6)$$

where the spatially correlated random effect ξ_i is a Gaussian random field with a distance based stationary Matérn covariance function given by

$$Cov\left(\xi(\boldsymbol{s}_i), \xi(\boldsymbol{s}_j)\right) = \frac{\sigma^2}{\Gamma(\nu)2^{\nu-1}} \left(\kappa \| \boldsymbol{s}_i - \boldsymbol{s}_j \|\right)^{\nu} K_\nu\left(\kappa \| \boldsymbol{s}_i - \boldsymbol{s}_j \|\right) \qquad (S7)$$

and $\| \boldsymbol{s}_i - \boldsymbol{s}_j \|$ is the Euclidean distance between locations $\boldsymbol{s}_i$ and $\boldsymbol{s}_j$; K_ν is the modified Bessel function of the second kind and order $\nu > 0$, which measures the degree of the smoothness of the process[1]; σ^2 is the marginal variance; and $\kappa > 0$ is the scale parameter.

Bayesian inference

The estimates of the model parameters were based on a Bayesian statistical inference approach, which was implemented using integrated nested Laplace approximation (INLA) in

conjunction with stochastic partial differential equations (INLA-SPDE[2,3]). Here, the use of the INLA-SPDE approach offers two key advantages: Firstly, it provides the Bayesian inference platform that allows for the use of prior knowledge and simplifies uncertainty quantification processes. Secondly, INLA-SPDE provides a computationally efficient alternative for computing the dense Matérn covariance function given in equation (7) by simply discretizing the entire spatial domain[2]. Finally, the predicted population count $\hat{C}_i$ is obtained as a product of the back-transformed population density $\widehat{D}_i$ and the settlement building count B_i, that is,

$$\hat{C}_i = B_i \, exp\left(\eta_i^{(D)}\right) \qquad (S8)$$

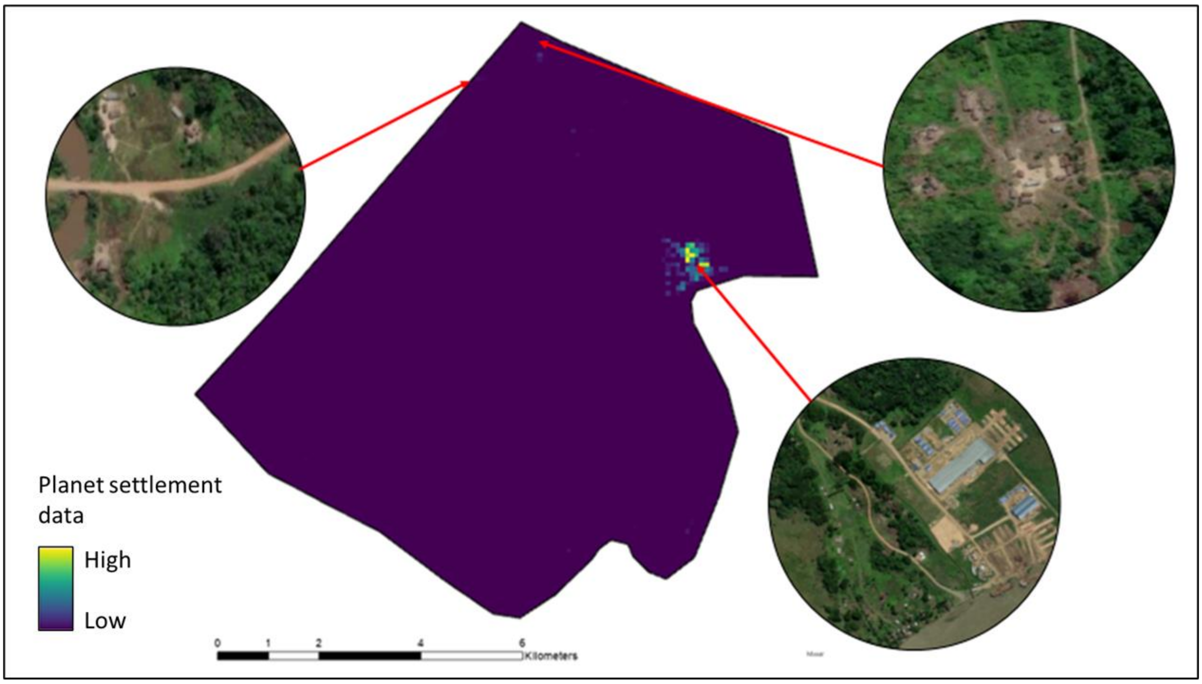

Figure S5. Example of biased Population predictions when settlement data are only partially observed in administrative unit Kanduanum 2. Satellite imagery sourced from ESRI.

Despite detecting more settled area than other more widely used satellite-derived settlement mapping datasets, the Planet settlement data picks up very little settled area in the unit showed above in Figure S5. As such, the observed settlements are disproportionally much smaller than is feasible for the observed population from the 2019-21 malaria survey. When divided by a disproportionately small number of buildings, the estimates of the population density, and prediction population count, become unrealistically high. For example, the 891 people identified within the census unit Kanduanum 2 were overestimated by about 7.5 times to 6690 when the BHM model was used. However, these were more accurately estimated as 897 people when the two-step approach was used.

Simulation Study - Methods

Data were initially simulated using the GPS points of the centroids of the 32,100 census units across the 24 Provinces in PNG (Figure S6). These include both the settlement building and population counts which were initially simulated as being completely observed at 100% level of observation, that is, the settlement building counts were ‘perfectly’ observed.

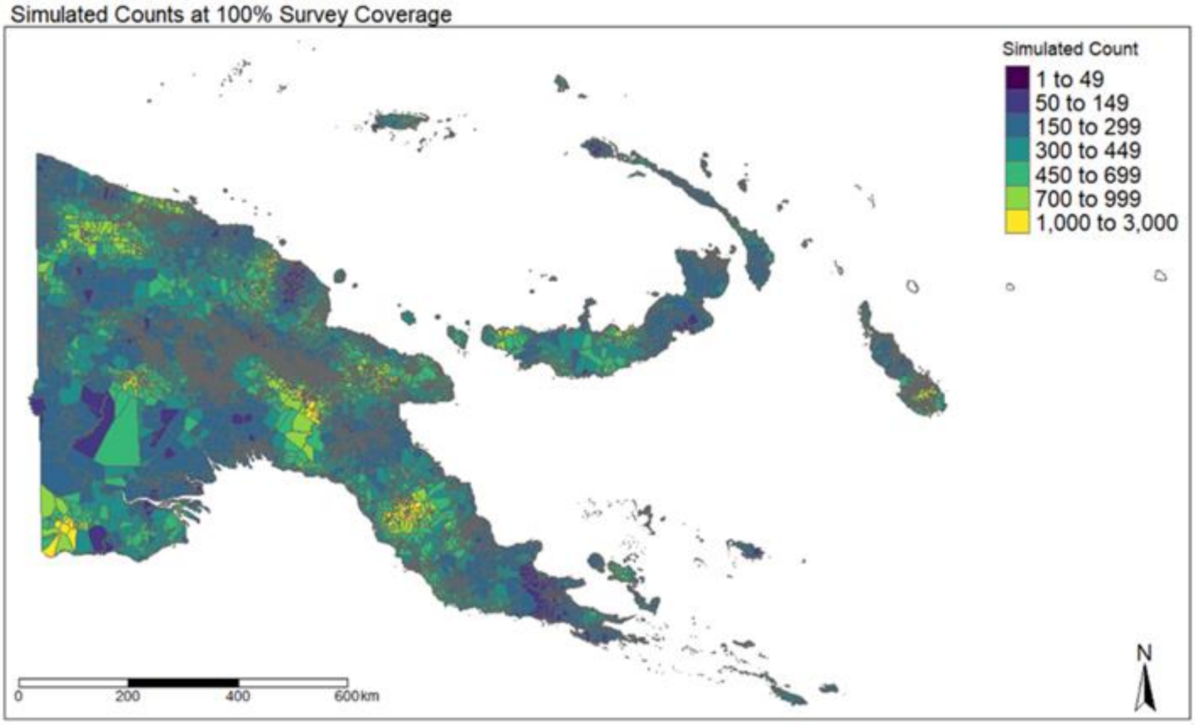

Figure S6. Counts of people across the 32,100 census units of PNG assuming 100% of the population and settlements were observed with a total population of 11,643,074 people.

Table S5. Simulation Study parameters

S/N	Parameter	Value
1	Smoothness parameter, ν	1
2	Range of spatial dependence, ρ	0.3
3	Marginal variance, σ^2	1
4	Scale parameter, κ	$\sqrt{8\nu}/\rho \approx 9.4$
5	Number of Mesh vertices	900
6	Coefficients of 5 geospatial covariates for simulating building intensity, $\boldsymbol{\beta}$	β_0=8.5, β_1=0.16 β_2=0.25, β_3=-0.21, β_4=-0.18, β_5=0.0935
7	Coefficients of 5 geospatial covariates for simulating Population count, $\boldsymbol{\beta}$	α_0=5.65, α_1=0.01 α_2=0.12, α_3=0.02, α_4=0.003, α_5=0.012

8	Initial number of Census Units	32100
9	Number of Provinces	24
10	Number of settlement types	3
11	Proportions of survey coverage	$p = \{0.2, 0.4, 0.6, 0.8, 1.0\}$
12	Proportions of Satellite observations coverage	$b = \{0.65, 0.70, 0.75, 0.80, 0.85, 0.90, 0.95, 1.00\}$,

The sensitivity of our methodology across the census units in the 3 settlement types of the 24 Provinces of PNG were tested for survey coverages from 20% to 100%, and satellite observation coverage of 65% to 100% such that a survey coverage of 100% with a satellite observation coverage of 100% means that the entire population were enumerated, and all human settlement structures were perfectly (unbiasedly) observed.

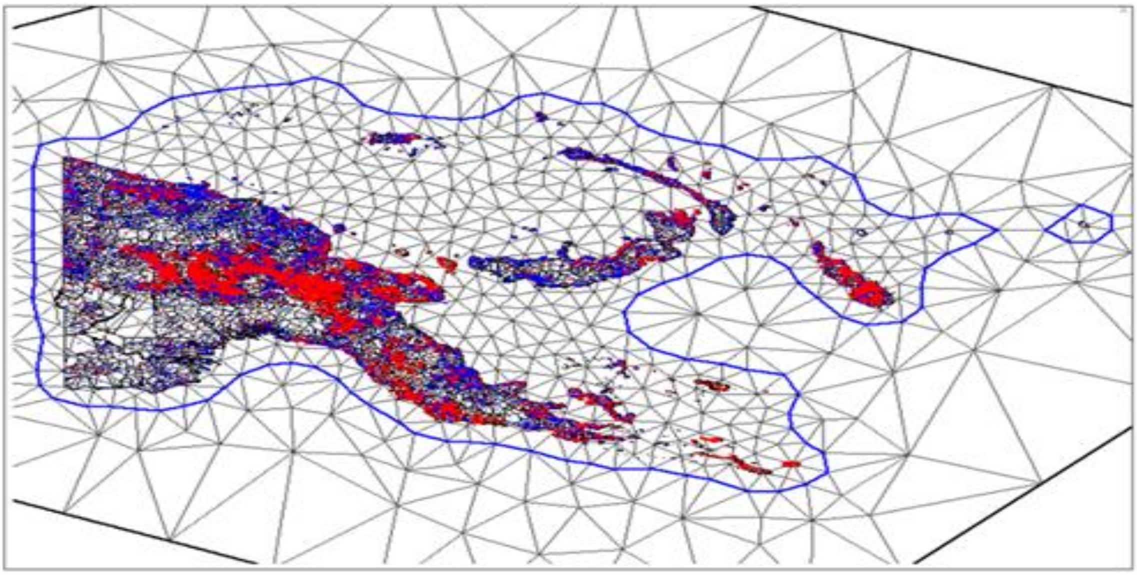

Figure S7. Non-convex hull mesh with 900 vertices (nodes) used throughout the simulation study. The red points are the centroids of the census units where the motivating datasets were observed. The mesh was constructed by first building the non-convex hull using the inla.nonconvex.hull() function and then creating the mesh by using the boundary argument of the inla.mesh() function with max.edge = (0.4, 4), and cutoff = 0.4. Full details of the mesh construction and the associated R codes are available on the GitHub repository here.

Below, we present the 8 key steps undertaken during the simulation study.

Simulation Study Steps

1) Specify the initial simulation parameters. The simulation parameters are presented in Table S5. Although most of these parameters are kept fixed throughout the study and only the parameters of interest (e.g., proportion of survey coverage p and proportion of satellite observation coverage b) are allowed to vary. Specifically, the SPDE parameters were chosen such that the scale parameter κ is approximately 9.4. The centroids of the census units were used to construct a non-convex hull mesh with 900 vertices across the entire 32100 census units (Figure S7). Then, we calculate the SPDE object and the sparse precision matrix $\boldsymbol{Q}(\boldsymbol{\psi})$ (where $\boldsymbol{\psi} = \{\kappa, \sigma^2, \nu\}$) and the projection matrix $\boldsymbol{A}$.

2) Simulate the geospatial covariates. After specifying the initial parameters and calculating the SPDE object and the precision and projection matrices, next is to simulate the geospatial covariates and then the building and population counts. The vectors of geospatial covariates $\boldsymbol{x}_1, \dots, \boldsymbol{x}_5$ are simulated from various arbitrarily chosen probability distributions:

$$\{\boldsymbol{x}_1, \boldsymbol{x}_5\} \sim Uniform(0,1)$$

$$\{\boldsymbol{x}_2, \boldsymbol{x}_4\} \sim Normal(0,1)$$

$$\boldsymbol{x}_3 \sim Poisson(2) \qquad (S9)$$

3) Simulate the building and population counts and then calculate the population density.

The building counts are simulated from a Poisson distribution with mean $\lambda^{(B)}$ given by

$$\lambda_i^{(B)} = \exp\left(\beta_0 + \beta_1 x_{i1} + \beta_2 x_{i2} + \beta_3 x_{i3} + \beta_4 x_{i4} + \beta_5 x_{i5} + \sum_{m=1}^{900} \boldsymbol{A}_{im}\tilde{\xi}_m + \zeta_i\right) \qquad (S10)$$

where β_0 is the intercept and $\tilde{\xi}$ is obtained from the SPDE object and ζ is drawn from a zero mean Gaussian distribution with variance parameter $\sigma_\zeta^2 = 0.05$. Then, the building count for the ith census unit is drawn from $B_i \sim Poisson\left(\lambda_i^{(B)}\right)$. Similarly, the corresponding population counts C_i are drawn independently from $C_i \sim Poisson\left(\gamma_i^{(C)}\right)$ such that,

$$\gamma_i^{(C)} = \exp\left(\alpha_0 + \alpha_1 x_{i1} + \alpha_2 x_{i2} + \alpha_3 x_{i3} + \alpha_4 x_{i4} + \alpha_5 x_{i5} + \sum_{m=1}^{900} \boldsymbol{A}_{im}\tilde{\xi}_m + \zeta_i\right) \qquad (S11)$$

where α_0 is the intercept and $\tilde{\xi}$ and ζ are being shared with the building counts.

Finally, the population density D_i of people per building is calculated as $D_i = C_i/B_i$.

4) Scale the covariates using Z-score (or mean centering) so that the model coefficients are interpretable in terms of standard deviations, where $Z_i = (X_i - \bar{X})/\sigma_x$, and $\bar{X}$ and σ_x are the corresponding mean and standard deviation of the covariate values.

5) Select the best fit covariates following a Generalised Linear Model (GLM) based stepwise regression based on the 'MASS' package. Use also the vif() function of the 'car' package to check for multicollinearity and retain only the covariates with vif values less than 5.

6) Specify and fit the INLA model using the inla() function of the R-INLA package

7) Carry out posterior simulation of the results to obtain more stable posterior estimates and then carry out model fit checks and cross validations.

8) Repeat steps 3 to 7 with different permutations of percentage survey coverage ($p\%$) versus percentage satellite observation coverage ($b\%$) so that altogether 40 different datasets were simulated and tested.

The total population simulated at each level of survey coverage ranged from 2,311,643 at 20% ($p = 0.2$) survey coverage to 11,643,074 people at 100% survey coverage (Table S6). Thus, the 'true' population is taken as 11,643,074 people and model performances are adjudged by high close the total population estimates are to the 'true' value across the various proportions of missingness. We are interested in knowing at what extent of missingness is our methodology able to recover the 'true' population and what impact does this have on the model parameter estimates uncertainty?

Table S6. Simulated total count of people across various

proportions of survey coverage

Survey Coverage ($p\%$)	Simulated/Observed total count
100	11,643,074
80	9,320,961
60	6,999,036
40	4,657,970
20	2,311,643

Furthermore, for each dataset, we first fitted the conventional BHM without correcting for the potential biases in the settlement data. Next, we fitted the TSBHM where we first adjusted for potential biases in the settlement data. Model performances were evaluated using a suit of statistical modelling fit indices (Table S7). In particular, we tested and compared the model performances and predictive abilities using the Mean Absolute Error (MAE), Root Mean Square Error (RMSE), Absolute Bias (Abias), Correlation Coefficient (CC), and Relative Error Rate (RER). Apart from the CC in which higher values indicate better fit, smaller values based on the other fit metrics indicate a better fit model.

Table S7. Model Fit Metrics

Metric	Equation
Mean Absolute Error (MAE)	$MAE = \frac{1}{n}\sum_{i=1}^{n} \lvert y_i - \hat{y}_i \rvert$
Root Mean Square Error (RMSE)	$RMSE = \sqrt{\frac{\sum_{i=1}^{n}(y_i - \hat{y}_i)^2}{n}}$
Absolute Bias (Abias)	$AB = \left\lvert \frac{1}{n}\sum_{i=1}^{n}(\hat{y}_i - y_i) \right\rvert$
Correlation Coefficient (CC)	$r = \frac{n\sum\hat{y}_i y_i - \sum\hat{y}_i\sum y_i}{\sqrt{\left(n\sum\hat{y}_i{}^2 - (\sum\hat{y}_i)^2\right)\left(n\sum y_i^2 - (\sum y_i)^2\right)}}$

For the MAE, RMSE and Abias, the smaller the better. While a higher CC value indicate a higher predictive ability.

REFERENCES

1. Florentin, J. J., Abramowitz, M. & Stegun, I. A. Handbook of Mathematical Functions. *The American Mathematical Monthly* (1966) doi:10.2307/2314682.

2. Lindgren, F., Rue, H. & Lindström, J. An explicit link between gaussian fields and gaussian markov random fields: The stochastic partial differential equation approach. *J R Stat Soc Series B Stat Methodol* (2011) doi:10.1111/j.1467-9868.2011.00777.x.

3. Rue, H., Martino, S. & Chopin, N. Approximate Bayesian inference for latent Gaussian models by using integrated nested Laplace approximations. *J R Stat Soc Series B Stat Methodol* 71, (2009).
